# The impact of inter-cycle treatment delays on overall survival in patients with advanced-stage ovarian cancer

**DOI:** 10.1093/oncolo/oyae201

**Published:** 2024-09-07

**Authors:** Luke Steventon, Kenneth K C Man, Shibani Nicum, Rowan E Miller, Shira Peleg Hasson, Samixa Shah, Michael Baser, Emma Kipps, Martin D Forster, Ofran Almossawi, Pinkie Chambers

**Affiliations:** Medical Oncology Department and Centre of Medicines Optimization Research and Education (CMORE), University College Hospitals NHS Foundation Trust, 235 Euston rd, London NW1 2PP, United Kingdom; Department of Practice and Policy, UCL School of Pharmacy, London WC1H 9JP, United Kingdom; Medical Oncology Department and Centre of Medicines Optimization Research and Education (CMORE), University College Hospitals NHS Foundation Trust, 235 Euston rd, London NW1 2PP, United Kingdom; Department of Practice and Policy, UCL School of Pharmacy, London WC1H 9JP, United Kingdom; Medical Oncology Department and Centre of Medicines Optimization Research and Education (CMORE), University College Hospitals NHS Foundation Trust, 235 Euston rd, London NW1 2PP, United Kingdom; UCL Cancer Institute, Department of Oncology, London WC1 6DD, United Kingdom; Medical Oncology Department and Centre of Medicines Optimization Research and Education (CMORE), University College Hospitals NHS Foundation Trust, 235 Euston rd, London NW1 2PP, United Kingdom; School of Medicine, Tel Aviv University, Tel Aviv, Israel; The Royal Marsden NHS Trust, London SW3 6JJ, United Kingdom; Medical Oncology Department and Centre of Medicines Optimization Research and Education (CMORE), University College Hospitals NHS Foundation Trust, 235 Euston rd, London NW1 2PP, United Kingdom; National Disease Registration Service (NDRS), NHS England, London E14 4PU, United Kingdom; The Royal Marsden NHS Trust, London SW3 6JJ, United Kingdom; Medical Oncology Department and Centre of Medicines Optimization Research and Education (CMORE), University College Hospitals NHS Foundation Trust, 235 Euston rd, London NW1 2PP, United Kingdom; UCL Cancer Institute, Department of Oncology, London WC1 6DD, United Kingdom; Great Ormond Street Hospital for Children NHS Foundation Trust, Population, Policy & Practice Department, London WC1N 1LE, United Kingdom; Medical Oncology Department and Centre of Medicines Optimization Research and Education (CMORE), University College Hospitals NHS Foundation Trust, 235 Euston rd, London NW1 2PP, United Kingdom; Department of Practice and Policy, UCL School of Pharmacy, London WC1H 9JP, United Kingdom

**Keywords:** cancer, ovarian, chemotherapy, treatment, delay, survival

## Abstract

**Introduction:**

Chemotherapy forms the cornerstone of systemic treatment for advanced ovarian cancer, extending overall survival; however, drug-related toxicity can lead to treatment delays, potentially diminishing treatment efficacy. This study evaluated the impact of treatment delays on all-cause mortality of patients with ovarian cancer, to better inform decisions on patient management.

**Methods:**

This retrospective, population-based cohort study included 1517 women with advanced-stage ovarian cancer, receiving first-line adjuvant or neoadjuvant chemotherapy in 2014 and 2015. The frequency of inter-cycle delays >7 days was calculated using drug administration dates. Kaplan-Meier estimates were used to compare 2-year overall survival (OS) between patients who were delayed and those treated to schedule. Cox proportional hazards regression was used to investigate the impact of treatment delay on all-cause mortality. Inverse probability of treatment weighting propensity scores were used to adjust for confounding variables.

**Results:**

Delays >7 days occurred in 35.3% of patients. Two-year OS probability was 62.7% in patients who experienced treatment delays >7 days (95% CI, 58.7-66.9) compared to 69.1% in those treated to schedule (95% CI, 66.2-72.0). Delays were not significantly associated with all-cause mortality when adjusted for confounders (HR 1.00 95% CI, 0.83-1.20, *P* = .9).

**Conclusions:**

Delays to chemotherapy treatment were not significantly associated with worsened survival in patients with advanced-stage ovarian cancer. These results can inform clinical decision making that prioritize toxicity management and quality of life for those treated with chemotherapy.

## Teaser

Chemotherapy for advanced ovarian cancer extends overall survival; however, drug-related toxicity can lead to treatment delays. This study evaluated the effect of treatment delays on all-cause mortality of patients with ovarian cancer, to better inform decisions.

**Implications for practice**

Delays between chemotherapy cycles occur frequently in patients with advanced ovarian cancer. However, prior to our work there was a lack of evidence on the association with these delays and overall survival. This research found no significant association (HR 1.00, 95% CI, 0.83, 1.20) that will reassure both clinicians and patients wishing to employ delays to manage toxicity.

## Introduction

Ovarian cancer is highly prevalent on a global scale, with an estimated 313,000 new cases recorded in 2022^1^ and around 7400 women diagnosed annually in the UK.^2^ Currently, no reliable screening test exists for ovarian cancer, and consequently most women are diagnosed at advanced stages (FIGO stages 3 or 4) after becoming symptomatic from their disease.^3^ Survival outcomes are poor in patients with advanced-stage ovarian cancer, with 2-year survival rates of around 41% for stage 3 patients, and 31% for stage 4 patients in the UK.^4^

Standard treatment for advanced-stage ovarian cancer involves cytoreductive (debulking) surgery followed by treatment with adjuvant or neoadjuvant systemic anti-cancer therapy (SACT) using platinum-based chemotherapy followed by interval debulking surgery.^5^ Treatment delays (between chemotherapy treatments) are often used to allow time for the patient to recover from toxicity and to maintain an acceptable quality of life (QoL). In advanced stages of disease, when survival outcomes are poor, there is a trade-off between increasing length of life through systemic treatment and maintaining QoL through minimizing drug toxicity. Treatments that are too aggressive can increase toxicity and the likelihood of additional hospital attendances, further increasing the burden of chemotherapy treatment for patients.^6^ Toxicity can be managed proactively by improving clinical monitoring and access to specialist clinics to address drug-related problems early in the course of an adverse-event, so that fewer patients require hospital admission for management of symptoms.^7^ Treatment delays will however be required for many patients to allow time for recovery from adverse- events.

Reports of the frequency of treatment delays in patients with ovarian cancer range widely, from 14.6% to 43.4% of treatments given during a standard chemotherapy regimen.^8,9^ The impact of delays on survival outcomes is, however, poorly understood, with some evidence suggesting that treatment delays can reduce survival through impaired dose intensity.^10^ Characterizing the impact of delays between chemotherapy cycles is essential to allow clinicians to make informed decisions about patient management, and to communicate with patients about the risks and benefits of delaying chemotherapy treatment. Several studies investigating the impact of delays in ovarian cancer have used small sample sizes or specific sub-groups, such as elderly patients, however this question has not yet been addressed in a large, population-based cohort.^11-13^

Further investigation into the association of delays with survival outcomes in advanced-stage ovarian cancer patients is warranted. This work aims to provide evidence that can inform approaches to optimize both chemotherapy treatment benefit and QoL for advanced stage ovarian cancer patients.

## Methods

This retrospective cohort study used population-based observational data from a large cohort of patients with advanced-stage ovarian cancer, in England, to quantify the frequency of inter-cycle delays occurring between chemotherapy cycles, and to investigate whether treatment delays were associated with overall survival outcomes.

### Data source

English registries that contained patient information for all patients with cancer treated with systemic treatment were available to conduct this study. The National Cancer Registration and Analysis Service (NCRAS) register all patients treated for cancer in NHS hospitals, providing information on surgery, diagnosis, age at initiation of chemotherapy, demographic information such as ethnicity socioeconomic status. These registrations were linked to the SACT dataset, also collected by NCRAS, which provides details on systemic treatment. These datasets were used to identify the patient cohort of interest using International Classification of Disease (ICD) codes specifying a diagnosis ovarian, fallopian tube, or primary peritoneal cancer of gynecological origin were included (ICD codes C48, C56, and C57).

### Study population

Data on adult patients with advanced-stage ovarian, fallopian tube, and primary peritoneal cancer of gynecological origin, receiving first-line adjuvant or neoadjuvant chemotherapy between Janaury 01, 2014 and December 12, 2015 in England were provided in the data request. Fallopian tube and primary peritoneal cancers were grouped with ovarian cancer based similar presentation and management. These cancers are collectively referred to here as “ovarian cancer.”

### Eligibility criteria and data handling

Patients with a diagnosis of stages 3 or 4 ovarian cancer were included. During the study period, standard of care for advanced ovarian cancer was 6 cycles of carboplatin and paclitaxel doublet treatment. Less fit patients may have received carboplatin single-agent according to British Gynecological Cancer Society guidelines, and therefore these patients were included in the study.^11,14^ Patients were excluded as follows: did not receive platinum-based chemotherapy, chemotherapy given within a clinical trial, treatment for synchronous cancer, missing drug administration data, treatment outside study period, missing death date information, surgery >1 year before start of chemotherapy treatment, diagnosis >6 months before chemotherapy treatment, patient received less than 6 cycles of chemotherapy. Cancer stages were grouped based on clinical guidance as “stage 3A/B, “stage 3C,” “stage 4.” Patients with incomplete staging data, listed as “stage 3” only (without further description) could not be assigned to 3A/B or 3C groups and were labeled as “stage 3.” Region and hospital type (local or academic) were assigned using NHS organization codes.

### Calculating delays to chemotherapy treatment

Delays to chemotherapy treatment were calculated using time between dates of drug administrations. Treatments were considered delayed when given >7 days later than expected based on standard drug regimen schedules.^12^ The definition of >7 day delay was chosen based on other studies of this kind^13,15,16^ that used this length of delay, as this period of treatment delay occurs commonly in patients receiving chemotherapy. Dates for interval debulking surgery (IDS) were used to exclude any delays resultant of surgery and associated recovery. Delays in bevacizumab administration were not classified as delays where this was used within a treatment regimen.

### Defining surgical status

Surgical status was categorized as primary debulking surgery (PDS) if they underwent surgery and started chemotherapy within 1 year. Patient whose surgery was performed up to 180 days after cycle one of neoadjuvant chemotherapy were identified as receiving interval debulking surgery (IDS).^17^ Patients who did not receive surgery were included in the study, as these patients represent an important subgroup that was recently highlighted in the Ovarian Cancer Audit of access to surgery in advanced ovarian cancer patients in England.^18^ Surgical status was incorporated into survival analysis as a covariate to account for these different treatment strategies.

## Statistical analysis

The outcome of interest was 2-year overall survival (OS). The outcome of 2-year OS was chosen as the primary outcome in concordance with other investigations of survival in advanced ovarian cancer^19^ with all-cause mortality being the outcome metric. Kaplan-Meier (KM) survivor functions compared survival between patients who experienced treatment delays and those who were treated to schedule. In this patient cohort, breaks in chemotherapy treatment following surgery are inevitable to allow for recovery from toxicity. To test whether there were disparities between English regions in this type of delay, descriptive statistics were used to compare time from surgery to next chemotherapy between regions.

Multivariable Cox proportional hazards regression was used to investigate the relationship between treatment delay and other covariates with OS. Inverse probability of treatment weighting (IPTW) propensity scores were applied to adjust for covariate differences treatment delay and treated to schedule patient groups.^20^ Covariates of interest were: age at start of treatment, cancer stage at diagnosis, body mass index (BMI), bevacizumab administration, chemotherapy regimen, surgical modality, Charlson comorbidity index (an index score of comorbidity based on ICD-10 codes), index of multiple deprivation, ethnicity and region, after a preliminary literature review identified these as variables of interest. Univariate linear and logistic regressions were used to test for associations between covariates and survival outcomes before incorporation into the Cox regression model. All data available were used, and a post-hoc sample size calculation was performed to confirm the sample size was sufficiently large to achieve 95% statistical confidence.

### Comparison of time for surgery break between regions

Time from debulking surgery to next chemotherapy treatment was calculated for each patient and compared between regions in England. Descriptive statistics were used to describe median time in days for each region to assess whether any regional differences exist in time to start or resume chemotherapy after debulking surgery.

### Missing data

Data were incomplete for some variables and labelled as “Unknown” where missing values could not be inferred from other available data. Height and weight values that were recorded erroneously were assigned a value of 0 and the patient’s BMI was labelled as “Unknown.” Multiple Imputation by Chained Equations (MICE) approach was used to impute estimated BMI values for patients where data were missing. Missingness of data is described in results.

## Results

### Description of cohort

**Figure 1.**
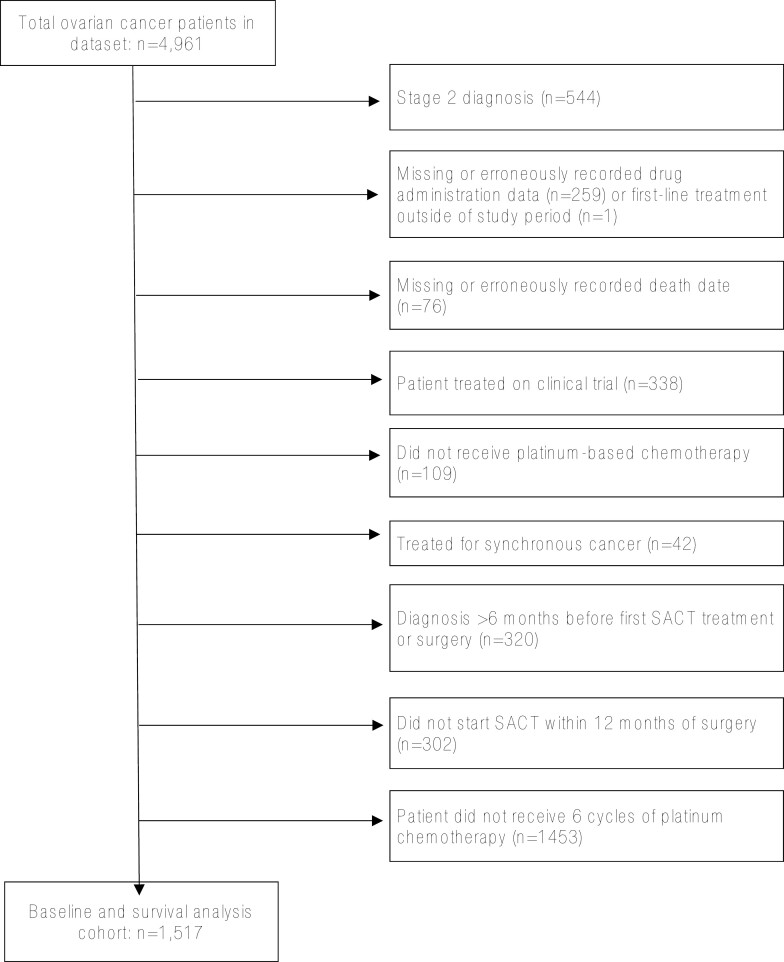
Exclusions made to extracted data.

A total of 1517 patients met inclusion criteria. Exclusions made are shown as a flowchart in Figure 1. Baseline characteristics of the study cohort are given in Table 1, showing overall characteristics of the entire cohort and a comparison of characteristics between patients experiencing treatment delay and those completing treatment to schedule. The median age was 66 years (range 18-91), with median age in the delayed group of 68 years compared to 66 in those treated to schedule. Supplementary Table S1 describes the study cohort stratified by surgical modality.

**Table 1.** Baseline characteristics of study cohort, showing total cohort and treated-to-schedule vs delayed cohorts (*n* = 1517)

Characteristic	Total (*n* = 1517)	Treated to schedule (*n* = 981)^1^	Delayed > 7 days (*n* = 536)^1^	SMD^2^
**Regimen**				
Carboplatin + paclitaxel (3-weekly)	985 (69%)	682 (69.2%)	303 (30.8%)	-0.008
Carboplatin + paclitaxel (weekly)	67 (4.4%)	30 (44.8%)	37 (55.2%)	0.003
Carboplatin (3-weekly)	460 (30%)	266 (57.8%)	194 (42.2%)	-0.002
Carboplatin (weekly)	5 (0.3%)	3 (60%)	2 (40%)	-0.001
**Age at first treatment** (IQR)	66 (58,73)	66 (57, 73)	68 (59, 75)	0.013
**Stage at diagnosis**				
3	95 (6.3%)	54 (56.8%)	41 (33.2%)	0.001
3A/B	204 (13%)	152 (74.5%)	52 (25.5%)	-0.015
3C	784 (52%)	511 (65.2%)	273 (34.8%)	-0.011
4	434 (29%)	264 (60.8%)	170 (29.2%)	0.022
**Surgery**				
Primary debulking surgery	499 (33%)	347 (69.5%)	152 (30.5%)	-0.016
Interval debulking surgery	540 (36%)	364 (67.4%)	176 (32.6%)	0.004
No surgery	478 (32%)	270 (56.5%)	208 (43.5%)	0.012
**Survival status**				
Alive	328 (22.8%)	228 (23.2%)	100 (18.7%)	-
Dead	1,187 (78.2%)	751 (76.8%)	436 (81.3%)	-
Unknown	2 (0.1%)	2 (0.2%)		
**Ethnicity**				
Asian	55 (3.6%)	34 (58.2%)	21 (41.8%)	0.016
Black	21 (1.4%)	14 (66.6%)	7 (33.3%)	0.003
Chinese	3 (0.2%)	3 (100%)	0	-0.051
Mixed race	5 (0.3%)	4 (80%)	1 (20%)	0.011
Not stated	28 (1.8%)	20 (71.4%)	8 (28.6%)	0.021
Other	17 (1.1%)	9 (52.9%)	8 (47.1%)	0.007
Unknown	5 (0.3%)	3 (60%)	2 (40%)	0.004
White	1,383 (91%)	894 (64.6%)	489 (35.4%)	-0.01
**Charlson comorbidity index**				0.014
0	1,357 (89.5%)	880 (64.8%)	477 (35.2%)	-
1	102 (6.7%)	61 (59.8%)	41 (40.2%)	-
2	34 (2.2%)	23 (67.6%)	11 (32.4%)	-
3	17 (1.1%)	11 (64.7%)	6 (35.3%)	-
4	7 (0.5%)	6 (86%)	1 (14%)	-
**Index of multiple deprivation**				
1—least deprived	336 (22%)	224 (66.6%)	112 (33.3%)	-0.005
2	356 (23%)	221(62.1%)	135 (37.9%)	-0.004
3	330 (22%)	225 (68.2%)	105 (31.8%)	0.006
4	283 (19%)	182 (64.3%)	101 (35.7%)	0.008
5—most deprived	212 (14%)	129 (60.8%)	83 (39.2%)	-0.005
**Bevacizumab treatment**	203 (13%)	131 (64.5%)	72 (35.5%)	-0.007
**Body mass index** (IQR)	25.5 (22.3, 29.4)	25.6 (22.3, 29.7)	25.4 (22.3, 28.8)	-0.013
Unknown	203 (13.4%)	113 (7.4%)	70 (4.6%)	

^1^ *n* (%); Median (IQR).

^2^Abbreviation: SMD, standardised mean difference of effect size between treated to schedule and delayed groups.

BMI was calculated for 1334 patients (87.9%) where height and weight data were available; for 183 patients (12.1%), missing height or weight values did not allow BMI to be calculated and were imputed using MICE.

### Frequency of treatment delays

Of the 1517 patients, 536 (35.3%) of patients experienced at least 1 delay to treatment of >7 days between chemotherapy cycles, excluding for breaks for IDS. Of all treatments given (*n* = 10 194), 9% (917) of all treatments given (*n* = 10 194) were delayed >7 days. Median length of delay was 15 days for 3-weekly regimens and 14 days for weekly regimens. In PDS patients, delays occurred in 30% of patients (*n* = 176), compared to 33% (*n* = 152) of IDS patients. In those who underwent debulking surgery, 44% (*n* = 208) of patients experienced treatment delays between either adjuvant or neoadjuvant chemotherapy cycles, accounting for breaks for surgery.

### Kaplan-Meier survival analysis

**Figure 2.**
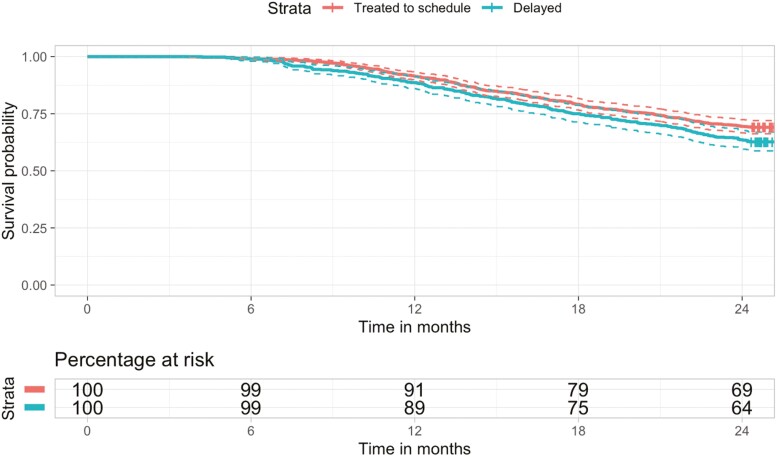
Kaplan-Meier survival curve comparing 2-year overall survival between patients who experienced inter-cycle delays > 7 days and those who were treated to schedule*.*

In total 1517 patients were included in KM survival estimates. Median follow-up time was 35.4 months. Two-year OS probability was 62.7% in patients who experienced treatment delays >7 days (95% CI, 58.7-66.9) compared to 69.1% in those treated to schedule (95% CI, 66.3-72.0), log-rank test *P* < .01. The KM curve comparing survival between groups is shown in Figure 2. Patients were censored at the timepoint of 2 years from initiation of chemotherapy if the event of all-cause mortality did not occur. Two patients had an unknown survival status at the end of the study period and were censored at the 2-year timepoint. Median OS was not reached and therefore not reported.

**Figure 3.**
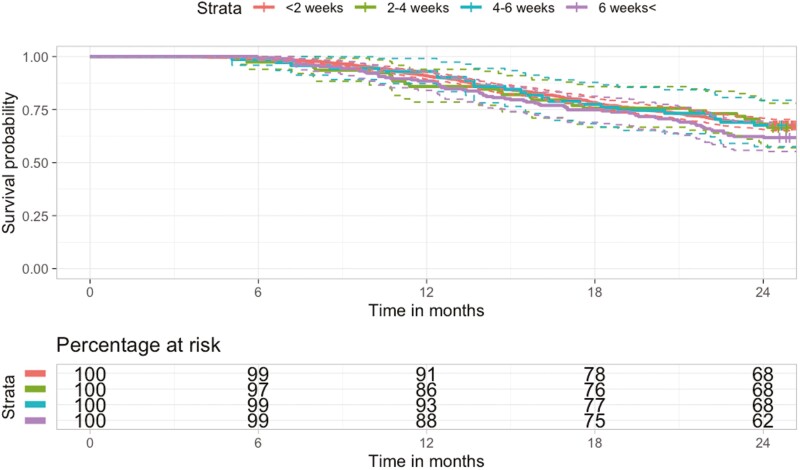
Kaplan Meier survival curve comparing 2-year overall survival between patients experiencing different lengths of treatment delay*.*

Figure 3 shows 2-year OS time by length of treatment delay. There were no significant differences in 2-year OS between patients who experienced <2 weeks, 2-4 week, 4-6 week, or > 6 week delay.

### Cox proportional hazards regression analysis

Table 2 describes the results of Cox proportional hazards regression analysis. A hazard ratio of 1.00 (95% CI, 0.83-1.20, *P* = .99) was calculated for the association of inter-cycle delays>7 days with the OS, adjusting for covariates using IPTW propensity scores. Covariates were well balanced between delayed and treated to schedule groups with all standardized mean difference (SMD) values within the 0.1 threshold, shown as a love plot in Supplementary Figure S1.

**Table 2.** Cox proportional hazards regression analysis.

Characteristic	HR^1^	95% CI^1^	*P*-value
Treatment delay > 7 days	1.00	0.83, 1.20	0.99

^1^  CI, confidence interval; HR = hazard ratio.

### Time from surgery to next chemotherapy treatment

**Figure 4.**
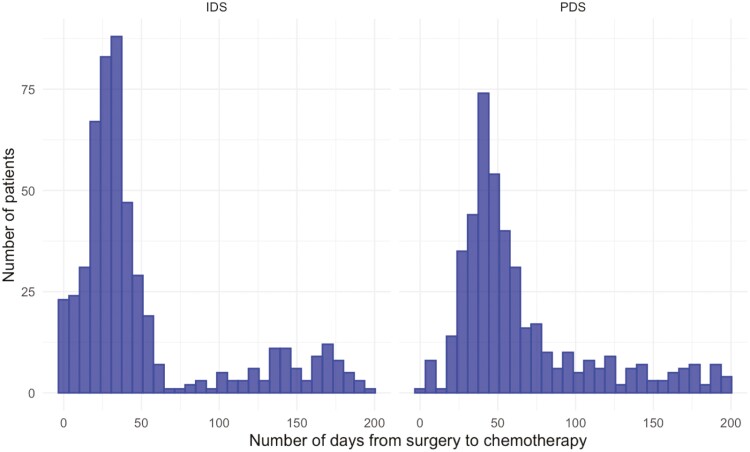
Histogram showing time from surgery date to date of next chemotherapy for patients receiving primary debulking surgery (PDS) or interval debulking surgery (IDS)*.*

Supplementary Table S2 shows median time from surgery to next chemotherapy by English region. Figure 4 shows time to next chemotherapy as a histogram, stratified into PDS and IDS cohorts. Time from surgery to next chemotherapy was comparable between regions for IDS patients (range 48-56 days). For patients undergoing PDS, time from surgery was markedly higher in the South East (91 days, IQR 70-143).

## Discussion

This retrospective study was the first study of its kind performed in a large, population-based cohort, finding no significant association of treatment delay with 2-year overall survival in advanced-stage, newly diagnosed ovarian cancer patients treated with platinum-based chemotherapy. This finding is a first indication that delaying chemotherapy treatment between treatment cycles is not detrimental to survival outcomes in this patient cohort and should reassure the clinical community.

In this study, on average 35.3% of patients experienced delays to chemotherapy. This frequency of delay is comparable to reports from other authors of 27%, 56%, and 28%-58%.^21-23^ The frequency of treatment delay during adjuvant and neoadjuvant chemotherapy was comparable between patients who underwent primary or interval cytoreductive surgery, but for patients treated with chemotherapy alone (without surgery), treatment delays occurred more frequently. This finding may be indicative of higher toxicity burden in this group, where surgery was not offered due to older age or poorer fitness. Median age of patients who did not receive surgery was 7 years greater than those who underwent PDS or IDS, which is concordant with reports of reduced offering of surgery to older ovarian cancer patients.^24^

The hazard ratio of 1.0 and CI of 0.83-1.20 suggests that treatment delay was not associated with survival after adjustment for covariates. These findings are concordant with evidence presented by others who have reported no significant differences in progression-free or overall survival between ovarian cancer patients who experience treatment delays and those completing chemotherapy regimens to schedule.^21^ Significant associations of treatment delay with poorer survival were not however reported in another study including only 184 patients (HR 1.75, 95% CI, 1.09-2.82, *P* < .05).^25^; Moreover, the authors reported a higher frequency of treatment delay (45.8%), than in this study, likely accounted for by inclusion of elderly patients aged 65 and over. Results presented here, using a large cohort of patients of all ages treated over a 2-year period in England, do not support this finding.

Other studies have investigated the impact of delay from diagnosis to initiation of treatment in patients with ovarian cancer, reporting small but significant associations of 1-2 month delay (HR 1.06, 95% CI, 1.01-1.11).^26^ A similar study, however, did not find a significant difference in survival between patients who started treatment immediately and those who had a >2 week delay before initiation of chemotherapy.^27^ As many ovarian cancer patients with ovarian cancer present at advanced stages of disease, prognosis is usually poor, with reports of median progression-free survival of 16-21 months and overall survival of 46 months.^24,28^ Toxicity management should therefore prioritize maintenance of acceptable QoL, as these results demonstrate that treatment delays >7 days were not significantly associated with survival.

Comparing time from surgery to next chemotherapy treatment showed that the surgical break period was comparable between regions of England for IDS patients. For PDS patients, hospitals in the Southeast region of England showed a slightly longer time for patients to receive chemotherapy after surgery. This finding warrants further investigation of causes related to increased waiting times for patients in this area. The impact of surgical break on survival outcomes was not investigated as part of this study as the exposure of interest was chemotherapy treatment delay.

Delays to chemotherapy can occur for many reasons, such as allowing time to recover from toxicity, hospital scheduling, and capacity issues and patient preference.^29^ The high frequency of treatment delays in this study, and reported by other authors, suggests that toxicity burden is high in patients with advanced-stage ovarian cancer. Findings from this study can support clinicians to prioritize toxicity management and QoL in patients with advanced-stage ovarian cancer being treated with platinum-based chemotherapy. Improved toxicity management strategies such as the use of Electronic Patient Reported Outcome Measures (EPROMS) may be applicable in patients with advanced-stage ovarian cancer, so that treatment-related toxicity can be managed at earlier stages to prevent escalation, requiring clinical intervention, or hospital admission.^30^ The time cost, financial burden and opportunity costs of undergoing chemotherapy treatment should also be considered within this context of poor survival outcomes.

The study period preceded the introduction of PARP-inhibitors as standard of care treatment, which were therefore not considered as part of this study. As the exposure of interest was platinum-based chemotherapy, this limitation does not impact the validity of findings presented. Histology and residual disease status following surgery are known to be important prognostic factors for survival in ovarian cancer,^31,32^ however these data were not available in the dataset and could not be included as covariates in survival analyses. Similarly, dose reduction and relative dose intensity could not be calculated as creatinine values were not available. Despite these limitations, this study benefits from the large cohort size and the availability of national population data, representing wide coverage of cancer treating hospitals in England.

## Conclusions

Our study demonstrated that inter-cycle delays days in standard first-line chemotherapy regimens were not significantly associated with 2-year overall survival outcomes in patients with advanced-stage ovarian cancer. Findings from this study can support clinicians to prioritize toxicity management and quality of life in ovarian cancer patients treated with platinum-based chemotherapy, rather than prioritizing completion of treatment to schedule despite high toxicity burden.

## Supplementary Material

oyae201_suppl_Supplementary_Material

## Data Availability

The data underlying this article will be shared on reasonable request to the corresponding author.

## References

[1] Huang J, Chan WC, Ngai CH, et al; on behalf of NCD Global Health Research Group of Association of Pacific Rim Universities (APRU). Worldwide burden, risk factors, and temporal trends of ovarian cancer: a global study. Cancers (Basel). 2022;14(9):2230. 10.3390/cancers14092230 35565359 PMC9102475

[2] Peres LC, Risch H, Terry KL, et al; Australian Ovarian Cancer Study Group. Racial/ethnic differences in the epidemiology of ovarian cancer: a pooled analysis of 12 case-control studies. Int J Epidemiol. 2018;47(2):460-472. 10.1093/ije/dyx252 29211900 PMC5913601

[3] Gupta KK, Gupta VK, Naumann RW. Ovarian cancer: screening and future directions. Int J Gynecol Cancer. 2019;29(1):195-200. 10.1136/ijgc-2018-000016 30640704

[4] Society, A.C., Survival Rates for Ovarian Cancer. 2018.

[5] Melamed A, Hinchcliff EM, Clemmer JT, et al Trends in the use of neoadjuvant chemotherapy for advanced ovarian cancer in the United States. Gynecol Oncol. 2016;143(2):236-240. 10.1016/j.ygyno.2016.09.002 27612977

[6] Gupta A, Eisenhauer EA, Booth CM. The time toxicity of cancer treatment. J Clin Oncol.. 2022;40(15):1611-1615. 10.1200/JCO.21.02810 35235366

[7] La Mola, L. , et al., 14 Enhanced supportive care–joint working between supportive care and acute oncology to deliver rapid access to expertise. 2018, British Medical Journal Publishing Group.

[8] Liutkauskiene S, Janciauskiene R, Jureniene K, et al Retrospective analysis of the impact of platinum dose reduction and chemotherapy delays on the outcomes of stage III ovarian cancer patients. BMC Cancer. 2015;15(105):1104-1105. 10.1186/s12885-015-1104-5 PMC4359455 25879527

[9] Starbuck KD, Szender JB, Duncan WD, et al Prognostic impact of adjuvant chemotherapy treatment intensity for ovarian cancer. PLoS One. 2018;13(11):e0206913. 10.1371/journal.pone.0206913 30418985 PMC6231633

[10] Nielson CM, Bylsma LC, Fryzek JP, Saad HA, Crawford J. Relative dose intensity of chemotherapy and survival in patients with advanced stage solid tumor cancer: a systematic review and meta-analysis. Oncologist. 2021;26(9):e1609-e1618. 10.1002/onco.13822 33973301 PMC8417866

[11] Early and locally advanced breast cancer: diagnosis and management, in National Institute for Health and Care Excellence (NICE) Guideline. London, United Kingdom: National Institute for Health and Care Excellence 2018. https://www.nice.org.uk/guidance/ng101.

[12] McNeil C. NCA *Breast cancer clinical guidelines, Northern Cancer Alliance*. 2018. **V2.10**. Accessed March 1, 2024. https://www.northerncanceralliance.nhs.uk/wp-content/uploads/2019/02/NCA-Breast-Cancer-Guidelines-v2-.10.pdf

[13] Schwenkglenks M, Jackisch C, Constenla M, et al Neutropenic event risk and impaired chemotherapy delivery in six European audits of breast cancer treatment. Support Care Cancer. 2006;14(9):901-909. 10.1007/s00520-006-0034-9 16622653

[14] BGCS, British Gynaecological Cancer Society (BGCS) Epithelial Ovarian / Fallopian Tube / Primary Peritoneal Cancer Guidelines: Recommendations for Practice. 2017, British Gynaecological Cancer Society: bcgs.org.uk.

[15] Gunasekaran GH, Hassali MABA, Sabri WMABW, Rahman MTB. *Impact* of chemotherapy schedule modification on breast cancer patients: a single-centre retrospective study. Int J Clin Pharm. 2020;42(2):642-651. 10.1007/s11096-020-01011-6 32185605

[16] Lyman GH, Dale DC, Friedberg J, Crawford J, Fisher RI. Incidence and predictors of low chemotherapy dose-intensity in aggressive non-Hodgkin’s lymphoma: a nationwide study. J Clin Oncol. 2004;22(21):4302-4311. 10.1200/JCO.2004.03.213 15381684

[17] Tangjitgamol S, Manusirivithaya S, Laopaiboon M, Lumbiganon P, Bryant A. Interval debulking surgery for advanced epithelial ovarian cancer. Cochrane Database Syst Rev. 2013;4(4):CD006014. 10.1002/14651858.CD006014.pub6 23633332 PMC4161115

[18] BGCS, BGCS Call to Action – Response to findings from National Ovarian Cancer Audit Feasibility Pilot. London, UK: British Gynaecological Society; 2021.

[19] Wang Y, Shan X, Dong H, Li M, Yue Y. Prediction for 2-year mortality of metastatic ovarian cancer patients based on surveillance, epidemiology, and end results database. Front Surg. 2022;9:974536. 10.3389/fsurg.2022.974536 36338661 PMC9632980

[20] Webster-Clark M, Stürmer T, Wang T, et al Using propensity scores to estimate effects of treatment initiation decisions: state of the science. Stat Med. 2021;40(7):1718-1735. 10.1002/sim.8866 33377193

[21] Nagel CI, Backes FJ, Hade EM, et al Effect of chemotherapy delays and dose reductions on progression free and overall survival in the treatment of epithelial ovarian cancer. Gynecol Oncol. 2012;124(2):221-224. 10.1016/j.ygyno.2011.10.003 22055764 PMC4035808

[22] Sivakumaran T, Mileshkin L, Grant P, et al; OPAL Study Group. Evaluating the impact of dose reductions and delays on progression-free survival in women with ovarian cancer treated with either three-weekly or dose-dense carboplatin and paclitaxel regimens in the national prospective OPAL cohort study. Gynecol Oncol. 2020;158(1):47-53. 10.1016/j.ygyno.2020.04.706 32381362

[23] Yi Q, Ran Y, Li C. The Effect of Delayed Chemotherapy on the Decrease of CA125 in Epithelial Ovarian Cancer During Coronavirus Disease Pandemic in 2020. Cancer Manag Res. 2021;13:515-520. 10.2147/CMAR.S289773 33500665 PMC7826069

[24] Viral P, Rajanbabu A, Pavithran K, et al Long-term survival outcome of advanced epithelial ovarian cancer: a single institutional study. Indian J Cancer. 2021;58(3):342-348. 10.4103/ijc.IJC_165_19 33402564

[25] Joseph N, Clark RM, Dizon DS, et al Delay in chemotherapy administration impacts survival in elderly patients with epithelial ovarian cancer. Gynecol Oncol. 2015;137(3):401-405. 10.1016/j.ygyno.2015.03.052 25839911

[26] Jing Zhao, R.C., Haiyan Zhu et al. PREPRINT Impact of treatment delay on the prognosis of patients with ovarian cancer: a population-based study using the Surveillance, Epidemiology, and End Results Database. Research Square (Preprint Version 1), 2023. 10.7150/jca.87881 PMC10758034 38169558

[27] Pyeon SY, Han GH, Ki KD, Lee K-B, Lee J-M. Effect of delayed palliative chemotherapy on survival of patients with recurrent ovarian cancer. PLoS One. 2020;15(7):e0236244. 10.1371/journal.pone.0236244 32701994 PMC7377458

[28] Paoletti X, Lewsley L-A, Daniele G, et al; Gynecologic Cancer InterGroup (GCIG) Meta-analysis Committee. Assessment of progression-free survival as a surrogate end point of overall survival in first-line treatment of ovarian cancer: a systematic review and meta-analysis. JAMA Netw Open. 2020;3(1):e1918939. 10.1001/jamanetworkopen.2019.18939 31922558 PMC6991254

[29] Kogan LG, Davis SL, Brooks GA. Treatment delays during FOLFOX chemotherapy in patients with colorectal cancer: a multicenter retrospective analysis. J Gastrointest Oncol. 2019;10(5):841-846. 10.21037/jgo.2019.07.03 31602321 PMC6776798

[30] Wintner LM, Giesinger JM, Kemmler G, et al Verwendung und Nutzen von Patient-Reported Outcomes in der onkologischen Behandlung: eine Übersicht. Wien Klin Wochenschr. 2012;124(9-10):293-303. 10.1007/s00508-012-0168-3 22538839

[31] Bryant A, Hiu S, Kunonga PT, et al Impact of residual disease as a prognostic factor for survival in women with advanced epithelial ovarian cancer after primary surgery. Cochrane Database System Rev 2022;2022(9):1465. 10.1002/14651858.CD015048.pub2 PMC9512080 36161421

[32] Yang SP, Su H-L, Chen X-B, et al Long-term survival among histological subtypes in advanced epithelial ovarian cancer: Population-based study using the surveillance, epidemiology, and end results database. JMIR Public Health Surveill 2021;7(11):e25976. 10.2196/25976 34787583 PMC8663583

