# Supplementary material for: The impact of inter-cycle treatment delays on overall survival in patients with advanced-stage ovarian cancer
Source: Oncologist. 2024 Sep 7;29(11):e1532–9. doi: 10.1093/oncolo/oyae201 (PMC11546639; doi:10.1093/oncolo/oyae201)

Supplementary Tables and Figure

Supplementary Table 1 – Baseline characteristics of cohort, stratified by type of surgical intervention

Characteristic	Primary debulking surgery N=499[1]	Interval debulking surgery N=540[1]	No surgery N=478[1]
**Delay >7 days**	152 (30%)	176 (33%)	208 (44%)
**Regimen**			
Carboplatin + paclitaxel (3-weekly)	364 (73%)	393 (73%)	228 (48%)
Carboplatin + paclitaxel (weekly)	16 (3.2%)	23 (4.3%)	28 (5.9%)
Carboplatin single-agent (3-weekly)	118 (24%)	122 (23%)	220 (46%)
Carboplatin single-agent (weekly)	1 (0.2%)	2 (0.4%)	2 (0.4%)
**Age at first treatment** (IQR)	64 (56, 72)	64 (57, 71)	71 (64, 77)
**Stage at diagnosis**			
3	27 (5.4%)	35 (6.5%)	33 (6.9%)
3A/B	130 (26%)	41 (7.6%)	33 (6.9%)
3C	272 (55%)	309 (57%)	203 (42%)
4	70 (14%)	155 (29%)	209 (44%)
**Survival status**			
Alive	174 (34.9%)	108 (20%)	46 (9.6%)
Dead	324 (64.9%)	431 (79.8%)	432 (90.4%)
Unknown	1 (0.2%)	1 (0.2%)	-
**Ethnicity**			
Asian	18 (3.6%)	19 (3.5%)	18 (3.8%)
Black	7 (1.4%)	6 (1.1%)	8 (1.7%)
Chinese	2 (0.4%)	0	1 (0.2%)
Mixed Race	4 (0.8%)	1 (0.2%)	0
Not stated	7 (1.4%)	8 (1.5%)	13 (2.7%)
Other	6 (1.2%)	6 (1.1%)	5 (1.1%)
White	455 (91%)	499 (93%)	429 (90%)

Characteristic	Primary debulking surgery N=499[1]	Interval debulking surgery N=540[1]	No surgery N=478[1]
Unknown	0	1 (20%)	4 (80%)
**Stage at diagnosis**			
3	27 (5.4%)	35 (6.5%)	33 (6.9%)
3A/B	130 (26%)	41 (7.6%)	33 (6.9%)
3C	272 (55%)	309 (57%)	203 (42%)
4	70 (14%)	155 (29%)	209 (44%)
**Charlson comorbidity index**			
0	454 (91%)	492 (91%)	411 (86%)
1	32 (6.4%)	32 (5.9%)	38 (7.9%)
2	10 (2.0%)	7 (1.3%)	17 (3.6%)
3	2 (0.4%)	6 (1.1%)	9 (1.9%)
4	1 (0.2%)	3 (0.6%)	3 (0.6%)
**Index of multiple deprivation**			
1 - least deprived	106 (21%)	127 (24%)	103 (22%)
2	110 (22%)	133 (25%)	113 (24%)
3	127 (25%)	104 (19%)	99 (21%)
4	91 (18%)	108 (20%)	84 (18%)
5 - most deprived	65 (13%)	68 (13%)	79 (17%)
**Bevacizumab treatment**	57 (11%)	89 (16%)	57 (12%)
**BMI**	24.8 (21.4, 29.0)	26.0 (22.7, 30.0)	25.6 (22.7, 29.3)
Unknown			

[1]*n (%); Median (IQR)*
[2]*Fisher's exact test; Wilcoxon rank sum test; Pearson's Chi-squared test*

Supplementary Table 2 – Median time from surgery to next chemotherapy administration by English region

		Number of days		
Surgery type	Region	Median	Q25	Q75
Primary debulking surgery	East of England	78.0	62.00	127.00
	London	71.0	59.00	161.00
	Midlands	78.0	63.00	134.50
	North East & Yorkshire	74.5	58.00	142.50
	North West	73.0	62.00	98.00
	South East	91.0	70.00	143.00
	South West	67.0	56.00	98.50
Interval debulking surgery	East of England	49.0	42.00	60.00
	London	48.0	38.00	56.75
	Midlands	56.0	43.75	67.50
	North East & Yorkshire	50.5	36.00	59.00
	North West	55.0	43.00	62.50
	South East	53.0	35.00	62.00
	South West	51.0	45.00	66.50

Supplementary Figure 1 – Love plot showing standardized mean differences after application of inverse probability of treatment weight (IPTW) propensity scores.

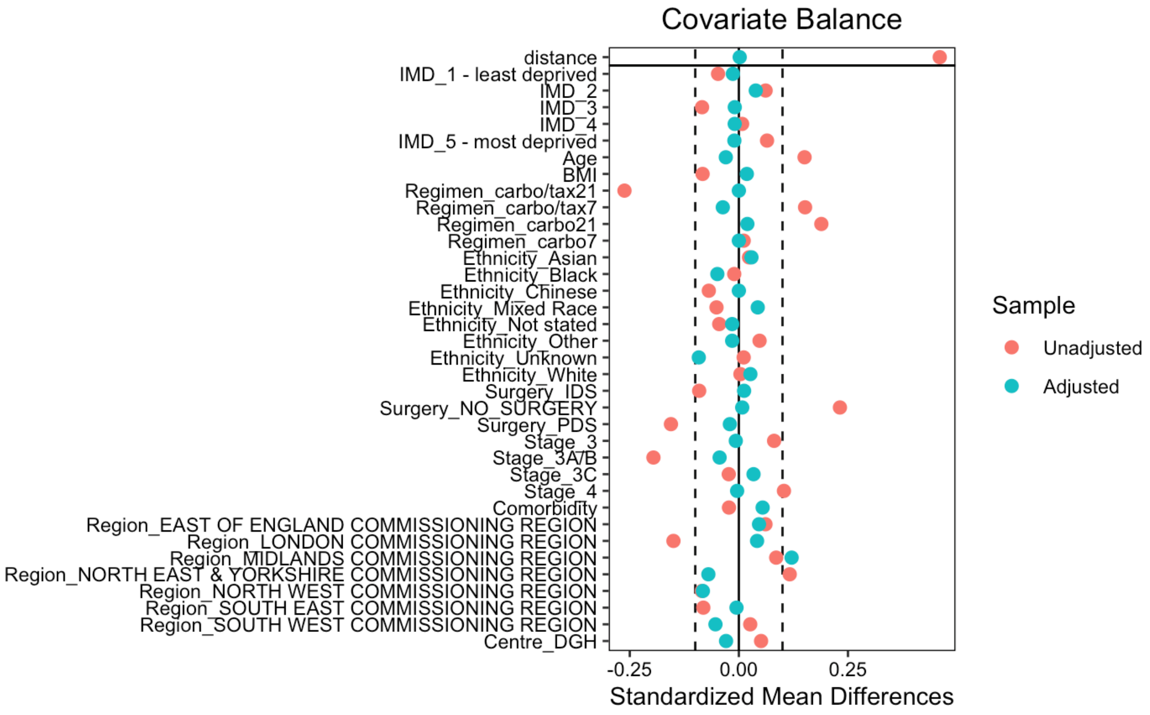

Supplement: oyae201_suppl_Supplementary_Material [file oyae201_suppl_supplementary_material.pdf]
